# One and done? Equality of opportunity and repeated access to scarce, indivisible medical resources

**DOI:** 10.1186/1472-6939-13-11

**Published:** 2012-05-24

**Authors:** Marco D Huesch

**Affiliations:** 1 USC Sol Price School of Public Policy, University of Southern California, Los Angeles, USA; 2 Leonard D. Schaeffer Center for Health Policy and Economics, Los Angeles, USA

## Abstract

**Background:**

Existing ethical guidelines recommend that, all else equal, past receipt of a medical resource (e.g. a scarce organ) should not be considered in current allocation decisions (e.g. a repeat transplantation).

**Discussion:**

One stated reason for this ethical consensus is that formal theories of ethics and justice do not persuasively accept or reject repeated access to the same medical resources. Another is that restricting attention to past receipt of a particular medical resource seems arbitrary: why couldn’t one just as well, it is argued, consider receipt of other goods such as income or education? In consequence, simple allocation by lottery or first-come-first-served without consideration of any past receipt is thought to best afford equal opportunity, conditional on equal medical need.

There are three issues with this view that need to be addressed. First, public views and patient preferences are less ambiguous than formal theories of ethics. Empirical work shows strong preferences for fairness in health care that have not been taken into account: repeated access to resources has been perceived as unfair. Second, while difficult to consider receipt of many other prior resources including non-medical resources, this should not be used a motive for ignoring the receipt of any and all goods including the focal resource in question. Third, when all claimants to a scarce resource are equally deserving, then use of random allocation seems warranted. However, the converse is not true: mere use of a randomizer does not by itself make the merits of all claimants equal.

**Summary:**

My conclusion is that not ignoring prior receipt of the same medical resource, and prioritizing those who have not previously had access to the medical resource in question, may be perceived as fairer and more equitable by society.

## Background

The efficient and fair distribution of scarce medical resources is one of the most difficult problems in society [1]. This intuitive trade-off between efficiency (e.g. medical need, ability to benefit) and fairness (e.g. waiting lists, equal chances) [2], may be further characterized as a trade-off between multiple core ethical values. Existing ethical guidelines on how to approach such allocation decisions are informed by many underlying moral principles [3]. These include treating all equally, favoring some on some basis, maximizing total benefits, and promoting and rewarding social usefulness (respectively, egalitarianism, prioritarianism, utilitarianism, and instrumentalism or reciprocity) [4].

For concreteness, consider the following example in the setting of allocating intensive care resources such as ICU beds. In the United States, the question of how to allocate limited resource is addressed in a critical care position statement authored by the specialty American Thoracic Society. The Society’s statement affirms the principle of egalitarianism in that: “Health care providers and institutions should ensure that ICU patients receive all of the resources that are medically appropriate to meet their needs” [5].

When resources are limited, the Society acknowledges that a situation might arise where “providing a disproportionate share of a health care system's limited resources to one patient may make resources unavailable to meet the needs of other patients. Problems of this sort represent a ‘tragedy of the medical commons’” [5]. While all life is valued, and valued equally, the guidelines recognize the question of the fairness of expending substantial resources on one patient and in so doing compromising the resources needed by others.

This implicit affirmation of a utilitarian principle might include the situation, for example, where “a patient with gastrointestinal hemorrhage continues to bleed despite all standard interventions and needs large amounts of blood products. When continued consumption of these blood products would jeopardize availability of blood supplies for others, it is justifiable for the institution to limit further transfusions” [5].

In far more resource constrained patient care settings, similar questions of allocation of scarce resources arise daily. Consider the Johannesburg hospital, where the clinical head of the department of pediatrics and child health described the impact of crushing resource constraints on ICU care allocation: “We don’t ventilate premature babies less than 1000 g. So a baby weighing 900 g has to take its chances without the support of a ventilator. About a third of them survive, but we could easily double that number if we were to ventilate them” [6]. Such a policy, in my view, could be justified by appealing to the principle of prioritarianism or utilitarianism. In the former case, priorities are being set based on clinical need and likelihood of benefit, while in the latter, the overall limited resources are being allocated to maximize the benefits across all infants who require skilled nursing care and ventilation equipment.

In this note I intend to focus narrowly on one particular application of the egalitarian principle of equality of opportunity to the allocation of health care resources. My narrow focus is on patients who have similar medical need for an absolutely scarce, indivisible medical resource. The amount of such resources is insufficient in aggregate to allow all to receive the resources. To break the ties between apparently equally-deserving claimants, resources are usually probabilistically allocated either through lotteries or time-based rationing of first-come-first-served [4,7,8]. I wish to challenge this, and suggest that the criterion of prior receipt of medical resources be used to prioritize against those who have received such resources before, all else equal.

The central question in this debate is thus whether equality of opportunity implies that all should receive equal priority on waiting lists for scarce organs, regardless of prior organ receipt. Conversely, the question is whether egalitarianism implies that all should receive the opportunity to have *one* organ. Informally, is it fair that some will have a second or third ‘helping’ before others have even had their first? For concreteness, I shall make use of a continuing example in the setting of organ transplantation: should we offer repeat kidney transplants to some retransplant candidates when other primary transplant candidates have not had their first.

I shall lay out the existing consensus arguments *for* ignoring past receipt of medical resources when deciding about future use of such resources. I intend to take issue with some of these arguments and will suggest a different approach to policy with regards to repeated use of scarce, indivisible resources. In particular, I intend to argue against ignoring past receipt of medical resources, and for prioritizing current allocations in favor of those who have not consumed such resources before, assuming medical need is otherwise equal. I also attempt to extend my perspective briefly to the use of only relatively scarce, divisible resources.

## Discussion

### Existing bioethical consensus

The bioethical consensus is that prior receipt of such scarce resources should *not* be a criterion for decisions about future distribution of the same resources [3,9,10]. Instead, the Council on Ethical & Judicial Affairs views the likelihood of benefit, the impact of treatment in improving the quality of the patient’s life, the duration of such benefit, the urgency of a patient’s condition, and the amount of resources required for successful treatment as all acceptable criteria for resource allocation.

In the continuing example of interest here, this consensus has been implemented. In the United States, the United Network for Organ Sharing, the private organization that managed organ waiting lists, follows the federal Department of Health and Human Services’ Final Rule for the Organ Procurement and Transplantation Network. The network therefore prioritizes kidney allocation based primarily on length of time on the waiting list, while other factors include whether the potential organ candidate is a child, body size of both donor and candidate, tissue match between donor and candidate, blood type, and blood antibody levels [11]. Similarly, in the Eurotransplant kidney allocation algorithm in Europe, the most important criteria are HLA matching, urgency, and waiting time. In particular, if failure of a graft occurs within three months, waiting time points are eligible for return, allowing the retransplant candidate to preserve priority status [12].

### Basis for existing guidelines

In the United States, these ethical guidelines rest on several arguments. The first is an argument made on the basis of insufficient and inconclusive guidance from common theories of justice and rejects consideration of prior receipt outright. The second, different, argument is that the principle of equality of opportunity requires only consideration of current needs, not past use. The third argument does not reject such consideration outright, but points to logical inconsistencies in how other receipt of non-medical goods is or is not considered.

Assuming similar health status, it has been argued that common theories of justice do not persuasively or consistently argue for or against considering prior receipt of resources [9]. In the absence of explicit guidance from society’s theories of justice, ignoring prior use of such resources would thus be a reasonable solution. In a seminal article in the context of organ transplantation, the main arguments Ubel and colleagues advance against considering prior resource use is that the conclusions of common theories of justice are neither persuasive (which is a weak, qualitative argument), nor consistent (which seems a far stronger and serious objection) when considering whom to favor [9]. Ubel and colleagues first ask whether favoring retransplant candidates over primary candidates could be justified, and find that fairness and morality do not favor this. They then ask whether the primary candidates should be favored over the retransplant, considering utlity, need, merit, age, social worth, ability to pay, personal responsibility and quality of life, and conclude that “these theories are therefore not much help in deciding how to treat retransplant candidates” [9].

This conclusion is supported by the position statement by the Council on Ethical & Judicial Affairs which also adduces another far more explicit argument for ignoring past use of medical resources. The Council argues: “the essential problem with a past use of resources criterion is that it rests on a fundamentally flawed conception of equality among potential recipients of treatment. Equality does not impose an ethical requirement that all patients receive the same amount of care; the only requirement is that patients be judged equally according to their current needs, based on their diagnoses and prognoses. Because past use is irrelevant to present need, it should not factor into allocation decisions” [3].

In my view, this argument already seems inconsistent with one of the Council’s ‘acceptable criteria for allocating medical resources’ which is the amount of resources required for successful treatment. The Council argues in the same position statement that “… occasionally, it may be appropriate to treat patients who will need less of a scarce resource rather than patients expected to need more. This would maximize the number of patients who could benefit from a scarce resource because each patient treated would require relatively little of it, thus making it more readily available for others” [3].

In my view, this utilitarian perspective appeals to the notion of maximizing the number of patients who could benefit from a scarce, divisible resource. But it seems easy to extend this notion of justice to one that would support my view of prioritizing access to scarce indivisible organs to those who had not yet received one. To do this conceptually, simply replace one divisible resource with a pool of indivisible resources, and contrast one patient receiving two units of those resources or two patients receiving one each.

A conceptually very different argument is why should society base allocation decisions on the narrow basis of whether such medical resources have been received before, without considering the patient’s prior receipt of or access to other medical or even social goods such as income, education or access to primary care? [9,10] These goods are clearly important as social determinants of health as “…neither the medical nor public health model is sufficient to improve the health of a population. Rather, improving health may require a wide range of strategies, including redistribution of wealth; guaranteeing all adults access to a meaningful job that pays an income sufficient to allow them to pursue healthy behaviors; helping children feel safe and be healthy and ready to learn; and empowering women and communities so that they can work more effectively to increase the health of the population” [13].

While access to such social goods can determine current health status, a more general argument can be made that fairness should involve consideration of prior access to such goods even without documenting a link to health. As Ubel and colleagues point out: “… the primary transplant candidate may have grown up with all of life’s advantages while the retransplant candidate has had to struggle to overcome poverty, absent parents, and inadequate access to healthcare” [9]. Their argument implies that principles of fairness would try to balance the receipt of all goods, medical or not, and impacting health status or not. This is clearly difficult to conceptualize let alone implement, and Ubel and colleagues stress the obvious conceptual and technical difficulties of trying to establish some sort of an allocation system that would consider prior consumption of multiple different medical and non-medical goods.

Accordingly the argument is then made that one should ignore prior receipt of all goods including prior receipt of past medical resource. This line of argument has been echoed recently by others who argue against the notion of a ‘fair share of resources’ in the context of age-based rationing. Rivlin argues that “we need to know the amount or size of what is being shared, the numbers of those among whom it is being shared among, and whether the claimants have any entitlement to it,” although he expresses his doubts as to our ability to satisfy those needs [10].

In my view, however, if we are unwilling or unable to even consider the first and simplest step in considering access to determinants of health (i.e. prior use of the medical resource under consideration), then we are implicitly and unpalatably giving up on more distant, less related and more complex determinants of health (e.g. social situation, income and asset levels, ability to interface with the health system, etc.).

The combination of these diverse arguments leads to the consensus that neither allocations which discriminate against prior recipients, nor ‘rule of rescue’ allocations which favor past recipients can be ethically supported. By the latter is meant a type of behavior and a rationale on the part of providers and their institutions which may feel a moral imperative to not abandon their patient, especially if it is a patient on whom they have operated or otherwise treated already, and even more especially if it is a patient they have done wrong by [14]. For example, in 2003 the Duke University Health System transplanted mismatched heart and lungs into a critically ill young female immigrant. Two weeks later, she was operated on again, given new correctly matched organs after the rejected organs were removed, ultimately and unfortunately to no avail [15].

In the prevailing views, all else such as medical need equal, only simple randomization without consideration of past access affords equality of opportunity. Ubel and colleagues find that only graft efficacy differences should allow us to favor primary versus retransplant candidates in the narrow context of organ transplantation [9]. The Council on Ethical & Judicial Affairs takes a far more general view, arguing against prior receipt of medical resources being considered for *any* intervention or care, and explicitly recommending first-come-first served as a randomization technique for allocation, all else being equal [3].

### Why prior resource receipt does matter

Yet this conclusion can be challenged on several fronts. While waiting time on a transplant list is seen as a seemingly fair and ethical allocation procedure and is in common use throughout the world, it is also true that different theories of distributive justice support different allocation criteria. For example, it has been pointed out in the setting of kidney allocation that there is no clear way within a system of normative ethics to decide on the balance between two criteria such as antigen matching and waiting time [16]. In my view, a criterion such as retransplant status may also be open to discussion and potential use to break ties when medical need is similar instead of using waiting time. In what follows I summarize three distinct strands of an argument against the conclusion of ignoring past receipt of medical resources. These span the views of other stakeholders, the informational value of prior receipt status, and the questionable comfort afforded by randomization.

### The views of other stakeholders

First, while some bioethicists believe that common theories of justice cannot justify considering prior receipt of resources, the views of those receiving, delivering, providing and funding such resources matter as well. Baily has argued more generally that bioethicists “need to understand the economic, political, medical and empirical dimensions of the health care rationing problem, to incorporate the insights of these fields into their theory…” [17]. Echoing this viewpoint, Ubel argues that the public deserves a role in decision-making or policy influence in setting treatment priorities. This is justified by the complexity of the solutions and the possibility that even expert philosophers can disagree [18].

In my view, there is substantial evidence that seeking such empirical data reveals important viewpoints. For example, in the United Kingdom, surveys among the general public on healthcare priority setting showed that respondents thought equality of access should prevail over maximization of benefits. For sufficiently effective treatments, it was preferred that hypothetical patients have a more equal chance of receiving treatment with less regard to their potential benefit from treatment [19].

In the context of the continuing example in this note, for example, patients with end-stage renal disease may or may not agree with existing guidelines. Surveys of the beliefs and opinions about kidney allocation procedures conducted among US end-stage renal disease patients find surprising misconceptions about the criteria determining priorities. Many thought that financial and quality of life status influenced position on the waiting list, while many African-Americans thought the system was biased against their race. Despite these considerations, few thought the system required revision, and the majority felt the current focus on antigen matching criteria was fair [20].

On the other hand, highlighting the importance of ascertaining opinions, other patients may disagree with allocation priorities. Interviewing a large number of hemodialysis and renal transplantee patients, a majority agreed with the current allocation priority for those who have waited longer. However significant proportions of patients were opposed to various aspects of the kidney allocation criteria, chiefly involving complex tradeoffs of antigen matching against time waited, whether dialysis had yet begun against time waited, and the use of age as a criterion. These findings led to a recommendation that patient opinions should be taken into account in designing allocation rules [21].

Surveys of medical students show similar differences in opinion about the fairness of repeated kidney transplantation. In Italy, students discussed a complex pediatric retransplant case and their preferences for allowing retransplantation were elicited under several framings. When there was no competition for allocation, 76% of students approved retransplantation, but when there were a total of 10 hypothetical patients vying for the scarce organ, only 32% approved retransplantion. When asked to imagine that they were the head of a Transplant department, 22% of students indicated they would change their opinions, further reducing support for allowing retransplantation [22].

Supply systems for absolutely scarce resources such as donor organs depend on the public’s trust that equitable measures will be used in the distribution. The World Health Organization Guiding Principle 9 states that “… in the light of the principles of distributive justice and equity, donated organs should be made available to patients on the basis of medical need and not on the basis of financial or other considerations” [23]. However it has been pointed out this does not address tie-breaking rules when medical need is similar but resources do not suffice for all. To cover such eventualities, it has been recommended that advocates who represent the community and the values of the society which ultimately furnishes the organs themselves should be involved in policy-making here [24].

The public’s trust in the principles used to guide such allocation and consent to the allocation rules seems especially important given that tax transfer payments ultimately fund much of the nation’s healthcare expenditures. It has been estimated that about 44% of all organ transplants in the United States are paid for by Medicare, with a further 9% paid for by Medicaid, making public monies the majority payor. In the US, end-stage renal disease implies eligibility for Medicare regardless of age, and Medicare will pay for the kidney transplants through its Part A and Part B programs. Still in the US, Medicare will also pay for pancreas, lung, liver, and in some circumstances, heart transplants. Medicaid rules vary by state but generally will cover kidney and liver transplants. Private insurance rules are complex but generally are at least as generous as the entitlement programs.

This role of tax transfer funding seems to make the inputs of the tax paying general public important too simply from a fairness perspective. For example, when choosing between different allocations of health gains, surveyed respondents not only consider efficiency, the total amount of health gains, but also equity, the distribution of the health gains [25]. Surveys have shown that primary recipients are favored for organ donations compared to those waiting to be retransplanted. Surveys of liver allocation rule preferences also reveal that the young are favored over the old; non-drinkers over drinkers, those more likely to survive, those who had waited longest on the list, and primary candidates over re-transplant candidates [26]. The most commonly stated rationale for these choices was given as prioritarian towards those most likely to survive and benefit from a liver transplant.

Other studies of preferences over liver allocation rules measured the relative importance people placed on prognosis and retransplantation status in allocating scarce transplantable livers. Respondents in the United States preferred to allocate scarce donor livers to those with better prognoses. This preference was slightly stronger among respondents in which prognosis was based on retransplant status than when it was claimed to be associated with a blood marker [27]. In this study, however, less than 1 in 5 respondents appeared willing to completely abandon the retransplant candidates by not allocating any organs to them.

Similar results were documented in the United Kingdom when university employees were queried about the relevance of the following criteria (% agreeing or strongly agreeing that criteria was important in the selection of liver transplants): age (66%), naturally occurring versus alcoholism-caused liver disease (72%), ability to survive and benefit (91%), waiting time on the list (63%), and primary versus re-transplant status (56%). Again, an overwhelming majority of survey respondents chose not to abandon the retransplant candidates [28].

In a broad-ranging literature review of this literature, community preferences for organ allocation schemes were reviewed from 15 studies [29]. These preferences were in conflict without broad consensus on how to balance criteria such as maximum benefit, social valuation, moral deservingness, prejudice, fair innings, first come, first served, and medical urgency. At least some community preferences were for a fair innings approach in which respondents felt that organs should be allocated based on the feeling that everyone is entitled to an equal chance at some “normal” span of health and thus give priority to younger patients. Relevant to this viewpoint, the community felt that all patients deserved at least one chance of transplant.

More generally, a preference towards trading off efficiency for more fairness in health care for has been noted in more abstract health care problems. Students were required to express preferences for one of two hypothetical societies, characterized by different distributions of life expectancy among hypothetical unfortunates (with shorter life expectancy) and hypothetical fortunate (with longer life expectancy). In framings in which the distributions were known as well as other framings in which there was uncertainty about the distribution, respondents preferred societies in which the life expectancy of the unfortunate short-lived individuals was improved at the cost of the fortunate longer-lived individuals. However, this preference was affected by the relative price of doing so in terms of how much life expectancy had to be sacrificed by the fortunate [30].

In related sophisticated experiments, samples of students and members of the general public were used to elicit generalized preferences for the trade-off between equity and efficiency in hypothetical health allocation situations involving cohorts of newborns. They found a strong aversion to inequalities in health [31]. Other work examining the trade-offs between efficiency and fairness has found a strong preference against allowing prior winners of probabilistic allocations of financial resources to participate in future draws [32].

In my view, despite the documented inconsistencies, this stream of literature lends some support to the role of stakeholders and their preferences in helping to shape allocation rules for scarce medical resources. The literature surveyed here reveals that conflicting opinions have been elicited regarding ethical principles that should be used, but there is scattered evidence that primary transplant candidates are somewhat preferred over retransplant candidates. I acknowledge that it is far from settled that such opinions should be elicited, listened to, let alone implemented. It has been argued that these small – possibly unrepresentative – public opinions about social value preferences may be worse than appealing to existing democratic processes that drive policy more formally [33].

### Informational value of prior receipt status

Second, while a narrow focus on a patient’s prior receipt of, say, an organ does ignore possible inequity in prior access to other non-medical resources, this does not justify ignoring that an organ was in fact received. In a strawman argument, society could equally well consider ever increasing sets of prior allocated medical or social goods [9]. It is implicitly accepted that ranking claimants on some measure(s) of past receipt of goods is possible, but difficult to implement. Depending on which goods were considered in such sets of goods, there might be different views of the fairness of medical allocation decisions. In the face of this difficulty, a seemingly neutral compromise is offered: ignore any and all information [9].

Yet such information on prior receipt can be simply and objectively assessed and is directly relevant to understanding the fairness of repeated receipt. Ignoring this information risks rejecting the essential along with the less essential, since such information is unlikely to have no value. Consider a thought experiment where a retransplant and a primary candidate of similar health status, age, and life experience are aware that both are vying for the same organ. If up to them, how plausible is it that a retransplant candidate would never consider letting a primary candidate have the next chance?

While this thought transplant is infeasible, a close practical analogy offers intriguing parallels and may inform the informational value of prior receipt status. Consider a healthy donor who forfeits his or her *own* existing organ so that another could benefit instead. For concreteness, assume a mother wishes to donate one of her own kidneys to a sick daughter. Of course, the example is not perfectly analogous, because in practice, if a candidate patient actually forfeited an organ, this would be seen as failure to cooperate with care, and could trigger refusal by the transplant network to offer future transplants [34].

Directed donations are ones in which a healthy donor states a preference for who is to receive the organ. Since there is a small risk of mortality, and significant morbidity from the living transplant procedure, there is rationally a cost from such altruism. Most common among healthy living donors directing a donation to a relative, they include donors who are not related to the ultimate beneficiary, and ones in which a donor donates to ‘the pool’ of organs [35]. Empirically, in the context of directed kidney donation, 80% of respondents expressed approval of the practice [36].

Some of these donations have been made for reasons that imply that some recipients are more special and more deserving than others, for example coming from the same town [37]. More morally repugnant versions would include expressed racial preferences. At least one example of a prima facie morally troublesome directed cadaveric donation has taken place in the organs of a white supremacist were used only for white recipients as per the deceased’s wishes [38]. Following this one-time example, the state of Florida passed a law banning these types of directed donation preferences [39].

Some views on directed donation diverge or are more nuanced. The UK’s Human Tissue Authority rejected the cadaveric donation of a daughter’s kidneys to her mother, since “the central principle of matching and allocating organs from the deceased is that they are allocated to the person on the UK Transplant waiting list who is most in need and who is the best match with the donor. In line with this central principle, a person cannot choose to whom their organ can be given when they die; nor can their family” [40]. This is troubling since the UK allows living directed donation. It seems that directed cadaveric donation is consistent with the former, while both are possibly inconsistent with the principle of greatest need [41]. A related opposing view holds that patients’ autonomy to direct donations posthumously is not in agreement with a purely egalitarian principle of justice [42]. However in an earlier different context the author held that living donors of gametes should have the right to direct their gametes to categories of recipients accepted as relevant by the moral or religious communities in their society [43].

However in general, directed donations appear to be compatible with existing bioethical principles of nonmaleficence, beneficence, utility, autonomy, and distributing benefits and burdens equitably [44]. It has also been pointed out that consistency implies that when “we permit living donation, we are in fact indirectly endorsing a form of directed donation” [45].

In my view, the apparent justice of allowing living organ donations directed to particular beneficiaries as opposed to arbitrary beneficiaries supports the importance of considering prior receipt of organs. Suppose we believe a mother is morally justified in sacrificing a kidney to a daughter who has effectively none. Then we may well also believe in the justness of allowing or guiding a patient who has already received a kidney to sacrifice his or her right to have another organ. Similarly, we may well also value allowing a donor to state a preference that his own kidneys be posthumously available to those who had not yet had a kidney transplant. To buttress the relevance of this analogy, almost half of kidney transplants today are via living donors [39].

Even if we are not willing to go this far, consider how ignoring the receipt of prior resources reduces the chance for altruism. Consider that while uninsured patients are unlikely to receive a needed organ transplant (comprising just 0.8% of all transplants), they are the source of as much as 17% of all organs [46]. Given the disproportionate incidence of important precursor illnesses, this suggests a profound sense of generosity, sacrifice and altruism already exists in this sector of healthcare. This altruism might be extensible to those who have had a turn on the waiting list and seen their graft fail. Considering their past receipt of resources and inquiring of their willingness to be altruistic and offer another a turn seems worthwhile. Completely ignoring the informational value of prior receipt of medical resources seems to go too far.

### The comfort of randomization?

The third difficulty with the current consensus is that in the face of such difficult, subjective decisions, randomization through lotteries or positions on waiting lists is held up as a neutral and safe solution. Yet randomization is a troublesome method of choosing which of two otherwise equal claimants is more deserving. While lotteries are generally held to be the least unfair mechanisms to allocate resources [47], this only holds true when all participants are equally deserving. Without clear agreement that this is actually true, use of a randomizer merely masks difficult choices that were not made. In particular, while under conditions of equal merit a randomizer seems warranted, the converse is not true. That is, the mere use of a randomizer does not by itself render all claimants equally deserving. Rather, it only renders all claimants equally likely to receive the resource in question.

These concerns are not theoretical: detailed empirical research shows that even when the use of a randomizer is deemed fair, it is not always thought to be appropriate [48]. A great reluctance has been found among experimental subjects to using randomization to choose between important and seemingly equivalent alternatives. The more serious the decision’s implications, the stronger the aversion. In a particularly relevant experiment on kidney transplantation, arguments to allot the kidney to one or the other of two patients were held to be equally strong and equally compelling by a slight majority (53%) of one group of respondents, yet a significantly smaller minority (26%) of a matched group of respondents chose to use a coin toss to decide between the two patients [48].

I also note that that the previously cited current ethical guidelines appeal to first-come-first-served as the effective randomization device. This probabilistic procedure is not theoretically different in its outcomes from a lottery or coin toss, but it has also been criticized for implicitly favoring the well-off, the better-informed, and those who have the resources to travel and queue quickly (e.g. without worrying about childcare or employment). For example, Apple founder Steve Jobs purchased a residence in the US state of Tennessee, and obtained his liver there. Tennessee is known to be a state with one of the quickest liver waiting lists in the nation.

In my view, the implicit comfort that policy-makers have with randomization as theoretically fair must be contrasted with the experimental evidence against such comfort. A decision-making process based on lot may thus not be accountable or reasonable, attributes which are held to be important in society’s decision-making on healthcare rationing and prioritization [49].

### A different model of prioritization

Given the concerns raised above, it is plausible that a more fair system of allocation of scarce medical resources would *not* ignore past receipt of such resources, and might prioritize against those with past receipt. Consider the continuing example of allocation of cadaveric kidney grafts. Historically, significant graft efficacy differences between primary and retransplant candidates were deemed the only valid reason for preferring primary to retransplant candidates [9]. However such efficacy differences have shrunk dramatically [50,51], due to better pretransplant screening and improvements in immunosuppressive medication. In consequence, a large and increasing proportion of patients awaiting a cadaveric kidney has had a prior kidney transplant [50]. Given that the kidney transplantation waiting list continues to grow [52], it is clear that allowing a previously transplanted candidate to receive a graft has real opportunity costs for a primary candidate.

Accordingly, I propose that when patients needing a transplant have similar medical needs, that retransplant status be used to prioritize patients for a renal graft. Instead of waiting time being used as the dominant criteria for prioritizing cadaveric kidneys, regardless of retransplant status, I propose a lexicographic allocation rule which considers medical need first, then retransplant status, and only then waiting time. The allocation rule I am suggesting is thus a combination of the prioritarian principle that underpins medical need as a criterion, and an adaptation of the egalitarian system which currently favors waiting time to provide equal opportunity to all on a list.

Under this rule, patients whose first graft failed would be obligated to choose between remaining on dialysis and going onto non-dialysis palliative therapy. In such an arguably fairer system, every primary transplant candidate has an increased chance of receiving a graft, and except for differences in medical need, no primary transplant patient would see a life-prolonging intervention be given instead to someone who had had such an intervention already.

Restricting such a basic tier to primary kidney transplantation should not be justified by efficiency – although this is certainly possible – but by perceived fairness. Indeed, focusing purely on an efficiency argument and a cost-effectiveness standpoint, repeat kidney transplantation already compares favorably with other common therapies [53]. In a strict utilitarian approach, decision makers should calculate the incremental cost effectiveness ratio for different potential treatments and allocate resources in increasing order from the lowest ratios representing the most affordable improvements in quality adjusted life years gained to the highest ratios representing the least affordable. Such an approach would also favor primary transplants, but I intend to focus on fairness arguments rather than appeal to utilitarian or efficiency arguments in this debate.

### Extensions of the framework

This framework could also potentially be extended to deciding between similar claimants on divisible and/or relatively scarce medical resources. I turn to this brief extension here. In Table 1, I sketch out a 2x2 matrix of scarcity and divisibility, highlighting the bulk of this paper’s focus on absolutely scarce, indivisible resources. By relatively scarce is meant resources and interventions whose supply is limited only by decisions on how many inputs to apply. More financial resources will allow a greater ICU infrastructure and more procured chemotherapeutic medication. By divisibility is meant the ability to take one common unit of the medical resource and use it for a greater number of patients than one. Of course these binary distinctions blur the practical boundaries: a liver is absolutely scarce, but could be divided to allow more than one patient to benefit. Blood products cannot easily be increased in supply, since donations are the critical input. However, increased costly marketing and a drive for autologous blood for example, could allow the supply to become less absolutely scarce.

**Table 1:** Scarcity and/or divisibility of healthcare resources

		**Divisibility of medical resource**	
		**Indivisible**	**Divisible**
Scarcity of medical resource	Absolute	E.g. *organ transplants*	E.g. blood products
	Relative	E.g. ICU beds	E.g. chemotherapy

I acknowledge that the current bioethical consensus is against considering prior receipt of any of the resources in Table 1, when decisions are to be made regarding current allocation. In the lower left cell, in the intensive care unit, the current ethical principles are that all lives are equally valuable, and it remains unethical to consider prior receipt of such medical resources [54]. However, prior receipt of resources, all else equal, could be used to prioritize resource allocation towards those who had consumed less intensive care in the past. It is theoretically possible to go from prioritizing kidney grafts to prioritizing intensive treatment of community acquired pneumonia in a geriatric population in an intensive care setting.

Analogous extensions to the right hand columns of Table 1 could be used to re-prioritize treatment for recurrent cancer below those for initial treatments. A partial implementation could simply be tiered co-payments that progressively increase a patient’s share of the cost of repeated treatment while holding patient responsibility for primary treatments at zero. Such an implementation would be analogous to the designs of value-based insurance that use tiered benefit structures to incent patient choices of more evidence-based and/or cheaper alternative pharmaceuticals. An unfortunate feature of such schemes is their regressive structure in favoring richer subscribers or patients for whom the relative cost is far smaller than for the poorer [55]. While not strictly denying repeated access to patients, such schemes could be seen as having unpalatable consequences on the distribution of healthcare.

### Relationship with a ‘decent minimum’

Prioritizing opportunities to receive a kidney transplant could also be seen to be part of a basic tier guaranteed to all and paid for by transfer payments. This would echo recent discussion on the ethical acceptability of tiered healthcare [56]. A related, earlier argument premised on the core value of equality of opportunity is that social resources such as healthcare are to be allocated so as to ensure that everyone has a ‘decent minimum’ and can attain the normal opportunity range for his or her society [57]. In Buchanan’s early view, the popularity of this notion hinges on a number of attractive features. First, “the idea of a decent minimum is to be understood in a society-relative sense. Surely it is plausible to assume that, as with other rights to goods or services, the content of the right must depend upon the resources available in a given society and perhaps also upon a certain consensus of expectations among its members” [57]. Clearly a societal debate is needed on the content of such a decent minimum [58], but in the context of organ transplantation, a decent minimum might well include only one kidney transplant because of the limited extent of available kidneys and perhaps because the expectations of society are such that one turn on a list for all is better than two turns for some.

Second, “the idea of a decent minimum is that since the right to health care must be limited in scope (to avoid the consequences of a strong equal access right), it should be limited to the 'most basic' services, those normally 'adequate' for health, or for a 'decent' or 'tolerable' life” [57]. Again, seen in the context of society’s expectations, the most basic service for those with end-stage renal disease would include free dialysis and could potentially include at most one attempt at kidney transplantation.

It is important to note that such a right to a decent minimum is difficult to support from a theoretical view or from the perspective of universal rights. It is argued that arguments from special-rights, such as the obligation to provide some standard of equal protection from particular harms, can contribute to supporting this notion of a decent minimum [57].

More recently, explicit rationing schemes have been proposed that implicitly reflect the fat-tailed distribution in consumption of healthcare and the finite resources of payors and funders. For example, in the United States currently, the top 1% of healthcare consumers consume 20.2% of all system healthcare expenditures and the top 15% consume 73.4% of all expenditures [59]. Given the resource constraints of payors and funders and the difficulties in access among under-insured and uninsured Americans, a reasonable approach to resource distribution is to postulate a right to a decent minimum of healthcare. Krohmal and Emanuel have noted that “… the principles of justice require society to provide its members with vital goods and services essential to human flourishing. Nonetheless, Rawls reminds us that the need for distributive justice arises precisely when scarcity precludes giving everyone all that they want or need. In allocating limited funds between competing public pursuits, justice’s demand that some critical services be provided is no less a requirement that other services of lesser importance or inordinate expense be forgone” [56].

### Relationship with a ‘fair innings’

In a type of age-based rationing, Williams popularized the concept of a ‘fair innings’ which can be related to the proposed prioritization scheme described in this note. His notion of a fair innings is predicated on the view that “while it is always a misfortune to die when one wants to go on living, it is not a tragedy to die in old age; but it is on the other hand both a tragedy and a misfortune to be cut off prematurely” [60].

Williams used this phrase as a cricketing metaphor for a fair length and quality of life – analogous to the number of runs scored in a single cricket innings. In an early contribution he pointed out that “… giving priority to one group of people means taking it away from another group… we must not shrink from identifying who (implicitly) the ‘low priority’ people are, in any particular system of health care” [61]. In his own view, the lower priority people should be those who like him who “should not expect to have as much spent on a health improvement for them as would be spent to generate the same benefit for someone who is unlikely ever to attain what we have already enjoyed. It calls for self-restraint by us elderly and especially by those of us who have flourished in health terms throughout our lives” [61].

The notion of a fair innings as a principle guiding healthcare resource allocation has been challenged. The prioritization of the young over the old in some settings would not be in line with Scandinavian government guidelines, for example [62]. Empirically, some support for the implied age-based rationing that underpins the fair innings argument has been found: respondents are willing to sacrifice overall health gains to favor the younger whose future or lifetime prospects are poor [63].

By contrast, the proposed approach in this note extends the idea of a ‘fair innings’ as a fair chance of treatment and, more specifically, a fair number of chances of treatment if the patient’s first treatment fails – analogous to the number of innings allowed. This notion of a ‘fair chance at bat’ is independent of, but related to a fair innings. Two patients in need of a particular scarce medical resource may have the same quality adjusted life expectancy, so the fair innings argument could be agnostic to who should be privileged in further allocation of medical resources. However, similar to the fair innings argument, a fair chance at bat argument reflects the same aversion to inequality, seeks to consider prior patient experiences, and is eminently and immediately quantifiable [64].

### Implications of proposed framework

More generally, allocation frameworks such as the one proposed here which favor those who had not previously accessed a particular resource could better align the incentives of patients with those of society. Knowing that an intervention was a ‘one off’ or would be personally more expensive next time could better incent recipients to adhere to medication and post-treatment plans. One might imagine in my continued example that a repeated transplant process is already a negative incentive, given the hospitalization, the disruption of usual life, the risks and the financial costs. However there is no evidence that the demand for repeated transplants declines. Indeed, waiting lists (including for retransplants) grow steadily and already far outpace the available supply of organs.

Knowing that a second treatment would cost the patient more or would not be allowed could similarly encourage a focus by patient and physician on evidence-based treatment the first time around. The possibility of such additional efficiency improvements over time would be further advantages of rethinking the current consensus against considering prior medical resource use. Without addressing the implementation challenges, such an approach would require that the ‘standard of care’ be changed by entitlement payors, private payors and specialist medical societies. In the United Kingdom, existing oorganizations such as the National Institute for Health and Clinical Excellence are successful exemplars of the implementation of such public processes that seek to “decide what new technologies, devices, or drugs should be part of the benefit package of the National Health Service” [65]. In the US, these efforts are far more diffuse, less well coordinated and underdeveloped. The implementation of the particular recommendation that I view positively in this paper, is unfortunately part of a much larger and more difficult discussion on how to ensure conformance with existing evidence-based guidelines [66].

Finally, I acknowledge that even successfully resolving this particular equality of access question does not necessarily address the potential for differences in quality and in health agency to still systematically impact different patients’ ability to achieve healthy functioning [67]. In the present context, it is possible that patients with prior use of medical resources have a systematically enhanced ability to interface with the care delivery system (i.e. they know how to ‘work the system’). All else equal, this could be yet another argument for weighting the scales against those patients in favor of more medically ‘naïve’ patients.

## Conclusion

Current bioethical guidelines hold that when allocating scarce, indivisible resources such as organs, no consideration should be given to retransplant status beyond its impact on medical need. Conditional on equal medical need, waiting time is the most important criteria for prioritization of kidney grafts. This allocation rule is thought to be equitable in that it affords equality of opportunity to all waiting. Based on a number of concerns, including empirically elicited social value preferences, I have suggested instead that conditional on equal medical need, primary transplant candidates should be given priority over retransplant candidates. A broader debate on existing allocation rules seems worthwhile given a universal the interplay between limited resources and growing need for scarce healthcare resources.

### Summary

The gap between what is possible and demanded in health care delivery, and what can actually be afforded is clear, already unbridgeable and still growing. While existing guidelines recommend against considering a patient’s past use of medical resources, when making decisions about the allocation of future resources, I have discussed several issues with this consensus. Most fundamentally, research on the preferences of patients and the general public suggests that such prior use should be considered when making decisions about future use.

The allocation framework sketched here prioritizes against those who are prior consumers of the scarce medical resource. It can be seen as improving the likelihood of all claimants receiving some absolutely scarce and indivisible resource, and reducing the chance that some receive more than one while others still wait. In my view, this could be part of a societally guaranteed ‘basic tier’ of benefits. More work is necessary to understand societal preferences, and the views of those who are not spectators but stakeholders in such allocation processes. However, it seems to me that from those to whom much has already been given medically, it may be time to ask something in return. For those who are missing out, getting a chance at a fairer turn seems worth more consideration.

## Competing interests

MDH discloses an award of £5,000 by the University of York’s Center for Health Economics through an *Alan Williams Health Economics Fellowship* aimed at further research of the approach described in this note. Dr Williams had proposed a related allocation system that prioritized those patients who had not yet lived a ‘fair innings.’

## Authors’ contributions

MDH is a former practicing community physician with MBBS degrees from the University of Sydney, Australia. He obtained his PhD in Policy at the Anderson Graduate School of Management at the University of California at Los Angeles.

## Pre-publication history

The pre-publication history for this paper can be accessed here:

http://www.biomedcentral.com/1472-6939/13/11/prepub
